# Local anatomy, stimulation site, and time alter directional deep brain stimulation impedances

**DOI:** 10.3389/fnhum.2022.958703

**Published:** 2022-08-03

**Authors:** Joseph W. Olson, Christopher L. Gonzalez, Sarah Brinkerhoff, Maria Boolos, Melissa H. Wade, Christopher P. Hurt, Arie Nakhmani, Bart L. Guthrie, Harrison C. Walker

**Affiliations:** ^1^Department of Neurology, The University of Alabama at Birmingham, Birmingham, AL, United States; ^2^Brainlab, Inc., Westchester, IL, United States; ^3^Department of Physical Therapy, The University of Alabama at Birmingham, Birmingham, AL, United States; ^4^Department of Electrical Engineering, The University of Alabama at Birmingham, Birmingham, AL, United States; ^5^Department of Neurosurgery, The University of Alabama at Birmingham, Birmingham, AL, United States

**Keywords:** Parkinson, deep brain stimualtion, impedance, directional DBS, orientation, subthalamic nucleus, anatomical localization, Brainlab

## Abstract

Directional deep brain stimulation (DBS) contacts provide greater spatial flexibility for therapy than traditional ring-shaped electrodes, but little is known about longitudinal changes of impedance and orientation. We measured monopolar and bipolar impedance of DBS contacts in 31 patients who underwent unilateral subthalamic nucleus deep brain stimulation as part of a randomized study (SUNDIAL, NCT03353688). At different follow-up visits, patients were assigned new stimulation configurations and impedance was measured. Additionally, we measured the orientation of the directional lead during surgery, immediately after surgery, and 1 year later. Here we contrast impedances in directional versus ring contacts with respect to local anatomy, active stimulation contact(s), and over time. Directional contacts display larger impedances than ring contacts. Impedances generally increase slightly over the first year of therapy, save for a transient decrease immediately post-surgery under general anesthesia during pulse generator placement. Local impedances decrease at active stimulation sites, and contacts in closest proximity to internal capsule display higher impedances than other anatomic sites. DBS leads rotate slightly in the immediate postoperative period (typically less than the angle of a single contact) but otherwise remain stable over the following year. These data provide useful information for setting clinical stimulation parameters over time.

## Introduction

Deep brain stimulation (DBS) is a remarkable therapy for neurological disorders, but the complexity of therapy is increasing with new device technologies such as directional leads. Implantable electrical stimulation represents a complementary tool to pharmaceutical treatments—very localized interaction with neural elements *via* a surgically implanted electrode array. Tissue impedance is a key property of electrode contacts on the implanted array, as it confirms electrical integrity of the system and impacts stimulation parameters that are required for effective therapy. Impedance is effectively the resistance within a given tissue medium and is fundamentally related to both the voltage and amount of current that can be delivered through the circuit.

Device technologies increasingly utilize more complex lead designs with greater numbers of contacts, directional selectivity, and considerations for sensing with future adaptive stimulation devices. Many currently commercially available devices now consist of electrode arrays far larger in length than the span of target brain structures leaving electrodes in adjacent brain structures. While electrodes outside target structures may or may not be used for therapy chronically, previous investigations have found impedances to vary by brain structure and over time with gradual decreases in electrode impedances over relatively long time intervals (Satzer et al., 2014, 2015, 2020; Wong et al., 2018). Electrode impedance should vary significantly among different anatomic sites since gray matter conducts electricity better than white matter (Laitinen et al., 1966; Latikka et al., 2001; Satzer et al., 2015). However, one group has measured higher impedance in gray compared to white matter (Satzer et al., 2015). The surface area of both the DBS contact(s) and the implanted pulse generator (IPG) contribute to the measured impedance, as well (Butson et al., 2006). Finally, prior work contrasting impedances of active versus unused electrode contacts showed trends toward lower impedances for contacts being used to deliver therapy (Hemm et al., 2004; Sillay et al., 2010).

Here we investigate both anatomic and longitudinal effects on the impedance of directional leads in the subthalamic nucleus (STN) region during the first year of therapy following DBS surgery for Parkinson’s disease. To determine anatomical properties of the leads, we localize the contacts in anatomical regions, and compare lead orientations during surgery, immediately after surgery, and one year later to measure potential directional rotation over time. We test four interrelated hypotheses. First, we hypothesize that directional contacts display higher impedance than ring contacts. Second, we hypothesize that impedances decrease over time. Third, we hypothesize that active stimulation decreases local tissue impedance. Lastly, we hypothesize that higher impedances are associated with closer proximity to white matter (internal capsule) compared to gray matter tissue.

## Methods

Participants were recruited as part of the SUNDIAL (SUbthalamic Nucleus DIrectionAL stimulation) study, a randomized, double-blind crossover study contrasting directional versus ring unilateral STN DBS for PD. Participants signed written consent prior to participation, and the STN target was recommended for routine care prior to recruitment for entry. Each participant was implanted unilaterally in the most severely affected brain hemisphere with Boston Scientific’s Vercise™ Cartesia 8-contact directional lead and Vercise™ PC IPG as part of the study under FDA Investigational Device Exemption G-170063. Surgical targeting was refined with awake multi-pass microelectrode recordings, macrostimulation, intraoperative imaging, and macrostimulation with the newly implanted DBS lead, as described previously (Bour et al., 2010). Based upon the final microelectrode recording trajectory, we set lead depth such that the dorsal STN border corresponded to the midpoint between the ventral and dorsal directional DBS rows (i.e., rows 2 and 3).

We measured lead positioning by co-registering pre-operative MAGNETOM Prisma MRI scans (Siemens Medical Solutions USA, Inc.) with intra-operative O-arm 2 CT (Medtronic, Inc.) and post-operative high-resolution CT (Koninklijke Philips N.V.) images in Brainlab (Munich, Germany). Brainlab software detects lead position and directional orientation and parcellates subcortical brain regions. Based upon CT artifact alone, lead orientation cannot be distinguished from the opposite orientation (180°). Following the approach of prior studies (Hellerbach et al., 2018; Dembek et al., 2019, 2021), we assumed the correct solution was the orientation closest to 0° (anterior facing), as intended by the implanting surgeon. However, one intra-op orientation of -85° was changed to +95°, which seemed more likely given that the subsequent post-op orientation measured +60°. A subset of participants elected to undergo staged DBS on the opposite side of the brain following their 12-month study exit visit. In these participants, additional CT images were obtained as part of routine care and exploited to remeasure lead orientation of the original DBS lead at longer follow-up intervals.

Standard triangular language (STL) files for each contact and anatomical region were exported from Brainlab. We loaded the STL files into MATLAB R2020a (MathWorks, Natick, MA, United States) to create 3D alpha shapes for each object (MATLAB functions “createpde,” “importGeometry,” “generateMesh,” “alphaShape”) (Figure 2A). We computed the percent of each contact’s volume inside each brain region of interest (MATLAB functions “inShape,” “volume”), and assigned each contact to the region with its most overlap. The regions of interest were subthalamic nucleus (STN), zona incerta (ZI), internal capsule (IC), thalamus (Th), and substantia nigra (SN). The percent volume of a bipolar pair of directional contacts in each region was computed as the average percent volume of the two contacts.

Electrode impedance (Ohms W) was measured for all DBS contacts during at least seven longitudinal encounters: stage 1 surgery after lead placement in the brain and prior to testing for efficacy, stage 2 surgery after implant of the extension wire and lead connection to the IPG, device activation approximately 1 month after implant, and at 2-, 4-, 6- and 12-months study visits post activation. Boston Scientific’s external trial stimulator and clinician programmer were used for all impedance measures. Only monopolar impedances were recorded during battery placement; otherwise monopolar and bipolar impedances were measured together. All measurements during DBS programming visits were performed prior to changes in DBS settings.

### Statistical analysis

Paired *t*-tests contrasted directional lead orientations at different time points. Linear mixed models (LMMs) tested four hypotheses regarding monopolar and bipolar contact impedance, using the “lme4” package in R Studio (Bates et al., 2015; R Core Team, 2020; R Studio Team, 2021). We utilized impedance as the dependent variable and included a random intercept by participant in all LMMs. First, to test whether directional and ring contacts had different impedances, post-operative monopolar impedances were pooled across visits, and we utilized contact geometry as a fixed effect (directional versus ring). Second, to test if monopolar impedances changed over time, we modeled directional and ring contacts separately with time as a fixed effect, using stage 1 surgery (lead placement) as the reference category. Third, to test the effects of time and active stimulation contact(s) on impedance we estimated models separately for monopolar and bipolar contacts that included a fixed interaction between time (days) and whether a contact was active or inactive. These analyses begin at the time of device activation and continue throughout subsequent study visits. Fourth, to estimate whether local anatomic tissue composition modifies impedance, we estimated the bipolar impedance of directional contacts (pooled across 1-, 2-, 4-, 6-, 12- month post-operative visits) using the percent volume of each contact pair in each region as fixed effects. We estimated the effect of local tissue with the bipolar impedances opposed to monopolar impedances since they provide a more local measurement.

## Results

### Lead orientation and localization

**FIGURE 1:**
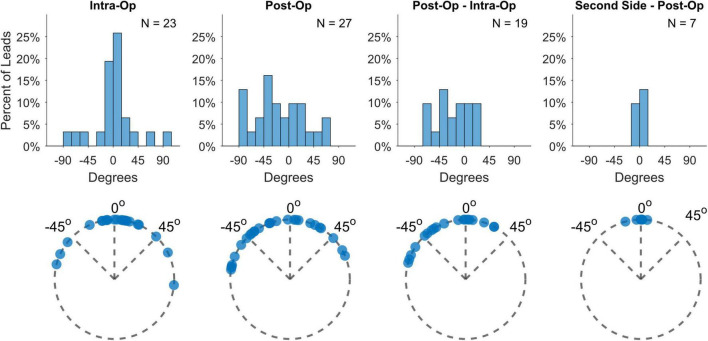
Lead orientation estimates during lead placement (intra-op) and immediately after surgery (post-op), as well as their differences and the differences from post-op to estimates derived from second-side surgery more than a year later.

**FIGURE 2:**
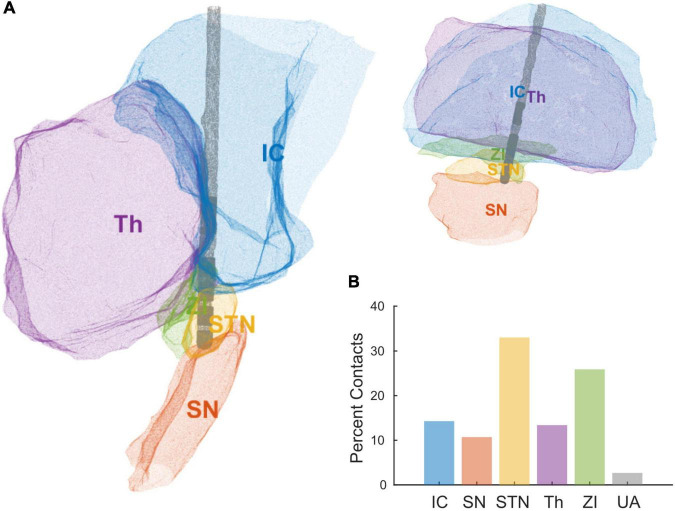
**(A)** Reconstruction of a single patient’s DBS lead and local anatomical regions using STL files exported from Brainlab. **(B)** Percent of contacts’ anatomical assignments, across all the patients, using the area of most overlap with a given a contact. Areas include internal capsule (IC), substantia nigra (SN), subthalamic nucleus (STN), thalamus (Th), zona incerta (ZI), and unaccounted (UA).

Deep brain stimulation lead orientations in intra-operative and post-operative CT scans were measured at 2.7° ± 37.2° (*n* = 23) and –16.5° ± 43.2° (*n* = 27) respectively, where 0° is the anterior direction based on the midsagittal plane and positive/negative angles are degrees lateral right/left rotation (Figure 1). The change in orientation from intra-operative scan to post-operative scan is statistically significant (–20.5° [CI: –36.3°, –4.8°], *p* = 0.013, paired *t*-test, *n* = 19) but considerably smaller than the total angular extent of a single directional contact (90°). A subset of participants (*n* = 7) underwent staged surgery on the opposite side of the brain after study exit which allowed assessment of potential changes in DBS orientation over longer time intervals. Orientation did not change significantly at these later time points versus the first postoperative scan (–1.5° [CI: –7.7°, 4.7°], *p* = 0.578, paired *t*-test). Based on the majority anatomic constituency per contact, most directional contacts localized to either STN (36.3%) or ZI (27.4%), however the location/composition of the more distant ring contacts were more variable (Figure 2B and Supplementary Figure 1).

### Impedance

**FIGURE 3:**
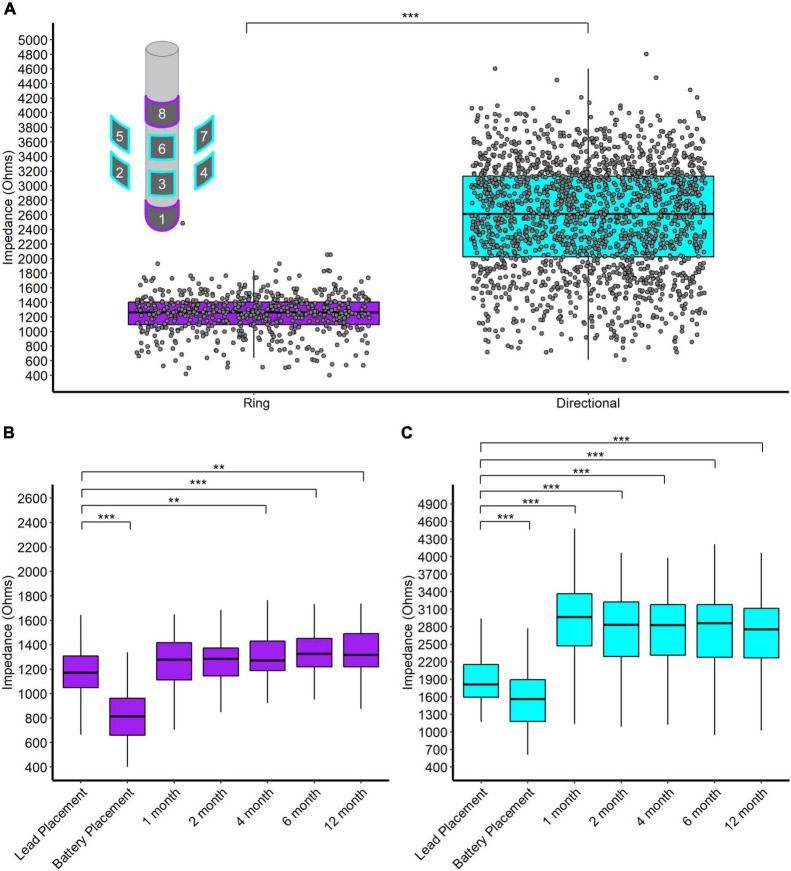
**(A)** Scatter and box plot of all monopolar impedance measurements (across patients, contacts, and visits) separated by ring and directional leads. Upper left contains a schema of the directional DBS lead. **(B)** Box plots of monopolar impedance for ring contacts across visits. **(C)** Box plots of monopolar impedance for directional contacts across visits. *p < 0.05, **p < 0.01, and ***p < 0.001.

We report four main results regarding impedance. First, directional contacts displayed significantly higher impedances than ring contacts, pooled across post-operative visits (2,559 and 1,242 W, respectively, *p* < 0.001, *n* = 32) (Figure 3A). Second, both ring and directional contacts had significantly lower impedances during stage 2 surgery (battery placement) than during stage 1 surgery (both *p* < 0.001, *n* = 32) (Figures 3B,C). However, impedances increased starting at 1-month versus stage 1 surgery for directional contacts (*p* < 0.001 for 1-, 2-, 4-, 6-, and 12-month follow-up visit each, *n* = 32, Figure 3C) and at 4-month for ring contacts (*p* = 0.004, *p* < 0.001, *p* = 0.001, at 4-, 6-, and 12-month follow-up, respectively, *n* = 32, Figure 3B).

**FIGURE 4:**
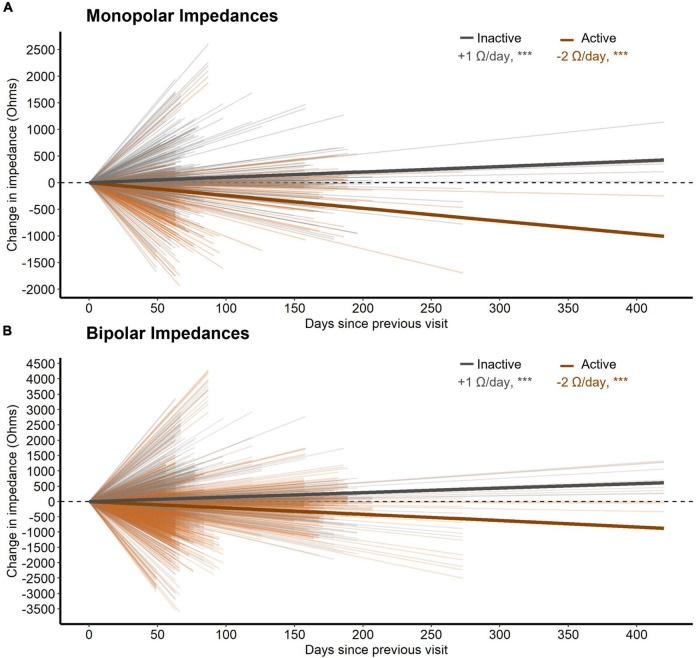
**(A)** The change in monopolar bipolar impedance per contact between subsequent post-operative visits as a function of time measured in days. Contacts are either inactive or active during this time. Thick lines represent the slope of impedance change for inactive and active contacts as determined by a LMM. **(B)** Same as **(A)** but for bipolar impedances. Here, a bipolar pair was labeled as active if either contact was active.

Third, active stimulation decreased both monopolar and bipolar impedances by 2.4 and 2.1 W/day respectively (both *p* < 0.001, *n* = 28, Figure 4). Conversely, inactive contacts displayed modest increases in both monopolar and bipolar impedances by 1.0 and 1.5 W/day, respectively over the 1-year follow-up interval (both *p* < 0.001, *n* = 28, Figure 4). The distance between bipolar pairs did not affect the change in impedance within a pair over time (*p* = 0.241, *n* = 28).

Finally, the local anatomic composition of a given pair of directional contacts significantly altered the measured impedance, such that for every 1% by volume of a bipolar pairing within internal capsule, the impedance increased by 4.2 W (*p* = 0.023, *n* = 27). In contrast, non-capsular structures such as STN, ZI, Th, or SN did not alter tissue impedance at our level of statistical power. As expected, distance between bipolar pairs increased impedance as well, such that every 1 mm increase in the distance between contacts increased impedance by 369.6 W (*p* < 0.001, *n* = 27).

## Discussion

While DBS lead orientations during surgery predominantly face anteriorly, a few leads displayed greater deviations than 60° rotation from the midline (17%), which is slightly higher than the 11% found in Dembek et al. (2019) Some additional rotation appears to be incurred between implant and immediately after surgery, probably related to lead fixation as one study found no additional rotation from the orientation measured from X-rays immediately after lead fixation [cite Kruger]. Interestingly, we measured a left bias for lead rotation during this time, despite the push-button design of lead fixation device. Unintended residual torque might be transmitted to the lead based upon the handedness of the surgeon or other factors. Nevertheless, the total rotation magnitude is usually relatively small and only rarely was the degree of rotation larger than the width of an entire directional contact. We measured no further rotation when scans were available more than a year later, consistent with a few studies which have used various methods for measuring orientation (Krüger et al., 2021; Lange et al., 2021) (Figure 1; Dembek et al., 2021).

Anatomic delineation of the local tissue environment in Brain lab and other related platforms appears to be useful in that DBS contacts can be linked more explicitly to local anatomy, without reliance on the ACPC coordinate system (Figure 2). These methods may prove useful for combining datasets across institutions in the future. Regardless, ACPC coordinates can be overlayed on the STL reconstructions if desired. This process is largely automated and provides greater anatomic specificity to guide targeting, postoperative programming, and analysis of intracranial electrophysiological signals. Of some interest, we measured greater tissue impedances in contacts most closely adjacent to the white matter of the internal capsule. Speculatively, the gradient of impedances within or across implant trajectories in an individual might be used as proxy for anatomic proximity to the internal capsule, a structure that can cause unwanted side effects in postoperative programming at the STN target in particular (Krack et al., 2002).

Directional DBS contacts showed higher impedances than ring contacts. The surface area of a directional contact is 1/4 of a ring contact’s surface area so we naïvely expected directional contacts to have 4 times the impedance of ring contacts. However, we observe that directional contacts have about twice the impedance of ring contacts on average (Figure 3). Likely, local tissue properties contribute further differences since directional contacts are more likely to be located in the STN/ZI area. Interestingly, impedances decreased at the time of battery placement but then trended higher during the first year after surgery, somewhat contrary to our initial expectations. The initial decrease in impedance might relate to temporary edema or effects of general anesthesia, while later increases over the first year may reflect resolution of edema, tissue scarring, and/or glial encapsulation. Thus, the accumulated evidence suggests modest increases in average impedance over the first year of surgery, likely followed by slower declines over much longer time intervals (Wong et al., 2018).

Contacts involved in chronic stimulation displayed relative decreases in tissue impedance versus inactive contacts, as well. Although distance between contacts changes the measured impedance substantially (Almeida et al., 2016), we did not note any effects of distance on its rate of change. Better understanding changes in electrode impedance over time has implications for understanding how the DBS electrical field may change over time within an individual. Similarly, changes in local tissue impedance could conceivably alter the recording environment for control signals for future adaptive stimulation paradigms. We are unable to comment on the clinical relevance here because, due to the blinded nature of this study, active contacts were changed throughout the course of the year making it difficult to interpret any effect of impedance change on stimulation settings for an given contact.

## Conclusion

Directional and ring contacts have different impedances but display similar longitudinal behaviors, with decreasing impedance immediately post-surgery followed by modest increases in over the first year. However, both active stimulation at a given site and longer follow intervals across all sites are associated with decreasing tissue impedances. Directional lead orientation can change modestly between intra-operative and post-operative scans, but otherwise appears to remain stable. Impedances within a given patient or recording site never completely stabilize, with different factors contributing to changes in the local tissue environment over time.

## Data availability statement

The datasets presented in this study can be found in online repositories. The names of the repository/repositories and accession number(s) can be found below: Data Archive for the Brain Initiative (DABI) at https://dabi.loni.usc.edu/home.

## Ethics statement

The studies involving human participants were reviewed and approved by University of Alabama at Birmingham IRB. The patients/participants provided their written informed consent to participate in this study.

## Author contributions

CG and HW: experimental design, data collection, and writing and editing the manuscript. JO: data collection, data analysis and/or making figures, and writing and editing the manuscript. SB: data analysis and/or making figures and writing and editing the manuscript. MB: data collection and edits of manuscript. MW, CH, BG: experimental design, data collection, and edits of manuscript. AN: experimental design and edits of manuscript. All authors contributed to the article and approved the submitted version.

## Supplementary material

Supplementary Figure 1 **(A)** Percent of contacts’ anatomical assignments, across all the patients, using the area of most overlap with a given a contact. Left and right bar plots are for directional and ring contacts, respectively. Panel **(B)** Similar to panel **(A)** but instead of the assigned region, plotted is the average percent volume of each contact contained in each region. Areas include internal capsule (IC), substantia nigra (SN), subthalamic nucleus (STN), thalamus (Th), zona incerta (ZI), and unaccounted (UA).
