# Supplementary figures and images for: Local anatomy, stimulation site, and time alter directional deep brain stimulation impedances
Source: Front Hum Neurosci. 2022 Aug 3;16:958703. doi: 10.3389/fnhum.2022.958703 (PMC9381736; doi:10.3389/fnhum.2022.958703)

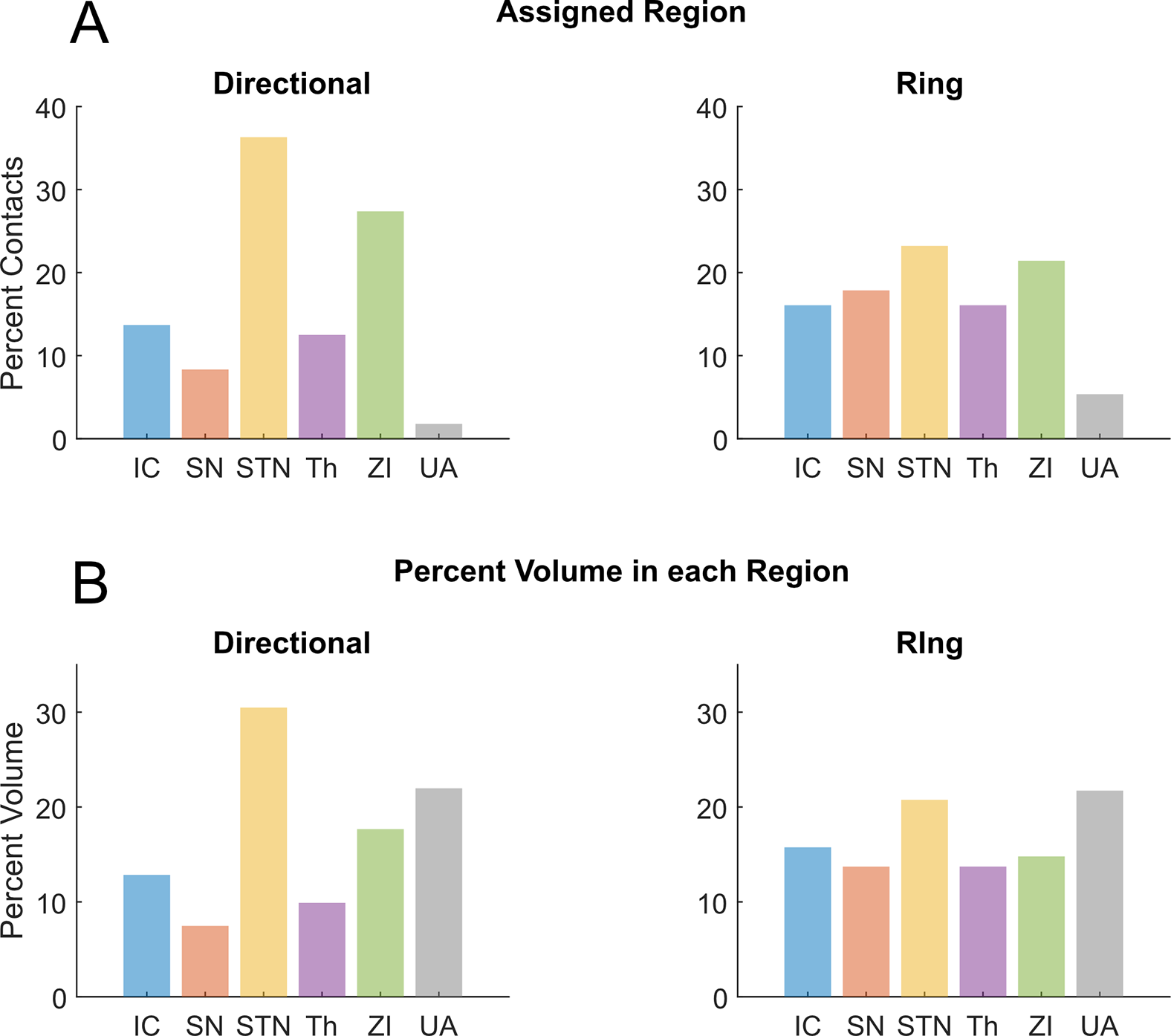

Supplement: Supplementary Figure 1 — (A) Percent of contacts’ anatomical assignments, across all the patients, using the area of most overlap with a given a contact. Left and right bar plots are for directional and ring contacts, respectively. Panel (B) Similar to panel (A) but instead of the assigned region, plotted is the average percent volume of each contact contained in each region. Areas include internal capsule (IC), substantia nigra (SN), subthalamic nucleus (STN), thalamus (Th), zona incerta (ZI), and unaccounted (UA). [file Image_1.tiff]
